# Affective Dimensions of Intergroup Humiliation

**DOI:** 10.1371/journal.pone.0046375

**Published:** 2012-09-28

**Authors:** Bernhard Leidner, Hammad Sheikh, Jeremy Ginges

**Affiliations:** 1 Department of Psychology, University of Massachusetts Amherst, Amherst, Massachusetts, United States of America; 2 Department of Psychology, New School for Social Research, New York, New York, United States of America; Boston College, United States of America

## Abstract

Despite the wealth of theoretical claims about the emotion of humiliation and its effect on human relations, there has been a lack of empirical research investigating what it means to experience humiliation. We studied the affective characteristics of humiliation, comparing the emotional experience of intergroup humiliation to two other emotions humiliation is often confused with: anger and shame. The defining characteristics of humiliation were low levels of guilt and high levels of other-directed outrage (like anger and unlike shame), and high levels of powerlessness (like shame and unlike anger). Reasons for the similarities and differences of humiliation with anger and shame are discussed in terms of perceptions of undeserved treatment and injustice. Implications for understanding the behavioral consequences of humiliation and future work investigating the role of humiliation in social life are discussed.

## Introduction

Which emotional state would you most dislike to experience yourself or to invoke in another person: anger, sadness, shame, or humiliation? We imagine that the feeling of humiliation would top the list for most readers. Humiliation, derived from the Latin humiliatus (made to lose self-respect) appears to have a strong aversive quality and to be significant across cultures; words that literally translate into the English “humiliation” and have the same connotation of lowering of status are found in languages as distinct as Hebrew, Polish, German, Hindi, Chinese and Urdu. Humiliation has been assumed to explain a variety of negative interpersonal and intergroup behaviors such as school related difficulties [1], psychological disorders [2]–[4], marital discord [5], domestic violence [6], poverty [7], as well as intergroup conflict and violence [8]–[18]. Despite its apparent real world importance across cultures, there is a paucity of empirical research into the experience of humiliation [19]–[20].

In this paper we report an empirical investigation into the emotional qualities of experienced humiliation. Against the background of frequent and large-scale injustices in ethnic or religious contexts, humiliation experienced due to an attack on oneself as a member of a social group appears particularly important. Thus, drawing on the intergroup emotion literature (e.g., [21]–[22]), we focus specifically on the experience of humiliation in an intergroup context. A substantial body of research exists investigating phenomena that are related to and can overlap with humiliation, such as hurt feelings as a consequence of social rejection (e.g., [23]–[24]), or emotional reactions to perceived insults of one’s honor (e.g., [25]–[27]). However, despite the relationship between these phenomena and humiliation, they have not been investigated in light of humiliation but rather in light of, for instance, anger and shame (e.g., [27]), or anxiety (e.g., [28]).

While there are numerous empirical studies on the emotions of anger, sadness, or shame, empirical studies that investigate or measure humiliation itself are surprisingly rare. Hartling and Luchetta [18] report the development of a scale measuring the cumulative impact of humiliation and fear of humiliation. Ginges and Atran [29] report studies investigating the effect of experienced humiliation on attitudes of Palestinians in the Israeli-Palestinian conflict, which led to less support for violence but also less support for peace deals. Combs, Campbell, Jackson, and Smith [30] asked participants to take the perspective of characters who had committed a moral transgressions in vignettes where the authors manipulated level of publicity and reprimand following the moral transgression. They found that levels of reported humiliation, anger, unfairness and vengefulness increased with levels of publicity and reprimand. Unlike the present study, however, they did not measure or manipulate individual experiences of humiliation. The present contribution adds to this emerging research on humiliation by manipulating experiences of humiliation, anger and shame in an intergroup context, investigating the extent to which these different emotional states were associated with feelings of outrage, powerlessness and guilt.

The near absence of empirical inquiry into humiliation may have led to a lack of clarity in discussions of humiliation and its role in social life. This is particularly problematic because people use humiliation to theorize about social phenomena, for example to explain intergroup violence (cf., [18]). On the one hand, humiliation and shame are often treated as synonyms in the literature (e.g., [31]). On the other hand, scholars seeking to link humiliation with violence treat humiliation as an extreme version of anger [19], [32]–[33]. This slipperiness may confound attempts to theorize cogently about humiliation, and to explain seemingly counterintuitive findings (e.g., [29]), as the emotions humiliation is frequently compared to and confused with (anger and shame) are associated with opposing behavioral tendencies. Shame is an inward facing emotion involving internal attributions of responsibility, leading to hiding, social withdrawal [34] and apologies and repair behavior [35]. In contrast, anger is an outward facing emotion, where another is deemed responsible for injustice [34], leading to a tendency towards aggression [36], to taking revenge and hurting the offender [35].

The primary goal of the present research was to investigate the affective characteristics of the experience of humiliation in intergroup contexts, and to compare the experience of humiliation with that of anger and shame. Our general expectation was that the experience of humiliation would differ systematically from the experience of shame and anger, respectively. That is, while the experience of humiliation was expected to overlap with anger and shame in some aspects (e.g. same level of powerlessness as present in shame, or same level of outrage as present in anger), we expected that its overall profile on all three aspects could be empirically distinguished from the overall profiles of both anger and shame. This could then help us understand why in some situations humiliation leads to hostility [30], but in other situations it leads to a state of inertia where people neither support violence nor peace deals [29].

We made three specific predictions regarding how experiences of humiliation would differ from experiences of shame and anger, respectively. Theoretical definitions of humiliation typically describe it as entailing the following: feelings of unjust degradation or devaluation in a social context [23], [37]–[38], with the individual perceiving his- or herself to be unable to respond to the degradation [18]–[19], [32], [39], [40]. As such, humiliation should be similar to shame in that both are social emotions that involve a sense of being less than one should be. Klein [1] has asserted that the distinction between the two emotions lies in the fact that ashamed people believe that they deserve their shame, whereas humiliated people feel that they do not deserve their humiliation (see also [40]). This theoretical notion is also suggested by more recent empirical findings that humiliation is associated with perceived unfairness [30]. This idea leads to the first specific prediction regarding discontinuities between experiences of humiliation and shame: if humiliation, in contrast to shame, is perceived to be undeserved, then experiences of humiliation should be associated with less feelings of guilt than experiences of shame [19], [37], [38], [41]. Being humiliated following a moral transgression [30] could also involve guilt, but likely because humiliation in that case should be more similar to shame and thus be perceived as more deserved, compared to the more general case of humiliation of non-transgressors, as investigated in our study.

Another difference between humiliation and shame lies in the situational aspects: Whereas shame can occur in private or in public, humiliation, it has been argued, is confined to public situations with an audience and a power asymmetry between ‘humiliator’ and humiliatee.’ Humiliation should thus lead to intense feelings of powerlessness (cf., [40]), at least as intense as the feelings of powerlessness typically involved in shame. If humiliation is related to feelings of degradation and powerlessness, but unlike shame also to feelings of non-deservingness, humiliated people might be more prone than shamed people to attribute blame for their negative experience to others rather than themselves (cf., [27], [42]–[43]). In this respect, and with respect to the aforementioned perception that one’s humiliation is undeserved, humiliated people should be more similar to angry than to shamed people. Therefore, according to our second prediction, they should experience greater intensity of other-directed outrage than shamed people.

The third specific prediction concerns the way in which humiliation may differ from anger. In the sense that humiliation involves feelings of rage in response to the unjust actions of others [20], [44], it appears similar to anger. Unlike anger, however, the experience of humiliation involves a loss of feelings of power and authority, which might be the reason why humiliation in intergroup contexts has been shown to lead to inertia rather than confrontation [29], despite humiliation leading to a *desire/motivation* for violence [30]. If you feel outrage toward the humiliator but at the same time you feel powerless, it is less likely that you will act on your outrage and engage in aggression and violence (cf., [1], [45]). To the extent that the loss of power is internalized in the experience of humiliation, it leads to a specific prediction regarding a discontinuity between humiliation and anger: experiences of humiliation will be associated with greater feelings of powerlessness than experiences of anger.

To test our predictions derived from the mostly theoretical humiliation literature reviewed above, we focused on the three dimensions implicated in our predictions in the investigation of the experience of humiliation and its commonalities with and differences from the experience of shame and anger: outrage (i.e., a specific type of anger provoked by the perceived violation of a personal or universal standard such as fairness; [46]–[48]), guilt (i.e., the feeling that one is at fault for an event; see for example [49]), and powerlessness (i.e., the feeling that one does not possess the necessary skills to respond to a problem or challenge; see for example [50]).

## Methods

### Ethics Statement

The study was approved by the institutional review board of The New School for Social Research. Written consent was obtained from all participants.

### Participants

Participants were recruited via the online portal Craigslist New York (www.craigslist.org) and the participant recruitment service Study Response Project [51]–[52], resulting in a more representative and heterogeneous sample compared to college samples. Twenty-four participants who could not remember which emotional situation they were asked to recall, and eight participants who, according to univariate outlier analyses [53] of the target emotions (humiliation, shame, anger), reported to feel the emotion they had been primed with to an extremely low extent (below the theoretical midpoint of the scale) were excluded from subsequent analyses. This left 213 participants (81.21% female, 18.79% male), with a mean age of 31 (SD = 10.44, range = 19–63), who were included in subsequent analyses. In all analyses reported below we investigated main effects of gender and sample (Craigslist vs Study Response Project) and whether either interacted with experimental conditions. No such effects were found.

### Procedure

Participants volunteered to take part in a study about emotions and minorities, and they were randomly assigned to one of three target emotions: humiliation, anger, or shame. To induce the emotional state we used the emotional event recall method [38], [54]–[57]. After self-identifying as a member of a social minority group they belong to (e.g., Blacks, Homosexuals, Muslims), the target emotion was induced by asking participants to remember and describe an idiosyncratic situation in which they felt the target emotion they were assigned to “in response to how someone treated you because you are a <member of minority group participant identified with>”. To measure the experience of each emotional state we then asked participants to indicate, in response to the remembered situation in which they felt humiliated, angry, or ashamed, as a member of a minority group, to which extent they felt each of twelve emotion words (all given in random order) aimed to capture the feelings of outrage (furious, upset, mad at others, offended), guilt (guilt, regret, remorse, sorry), and powerlessness (nervous, helpless, exposed, weak). Next, participants answered demographic questions regarding country of birth, gender, and age. Before being thanked and debriefed, participants also reported to what extent they felt humiliated, ashamed, and angry, presented in random order and later to be used as manipulation checks. All ratings were given on visual analog scales with the endpoints not at all (1) and very intensely (9).

## Results

### Manipulation Checks

To see whether our priming of emotions was successful, we ran planned contrasts, comparing the average intensity of the primed emotion (for example, the emotion word humiliated for those primed with humiliation) with the average intensity of the relevant emotion word for the other two groups combined. As all our hypotheses in this regard were directed, one-tailed tests were used.

Participants in the humiliation prime condition reported more intense feelings of humiliation (M = 7.86, SD = 1.20) than participants in the shame (M = 7.30, SD = 1.71) and anger conditions (M = 6.19, SD = 1.62), F(1, 206) = 15.77, p<.001, η^2^ = .07, d = 0.68. As some of the mean differences, despite their significance, are small, besides η^2^ as an indication of the explained variance we also report Cohen’s d as an indication of the magnitude of the mean differences. Participants in the anger prime condition reported more intense feelings of anger (M = 7.77, SD = 1.23) than those in the shame (M = 7.19, SD = 1.95) and humiliation conditions (M = 7.17, SD = 2.30), F(1, 207) = 5.13, p<.05, η^2^ = .02, d = 0.39. Finally, participants in the shame prime condition reported more intense feelings of shame (M = 7.41, SD = 1.26) than those in the anger (M = 3.63, SD = 2.52) and in the humiliation conditions (M = 4.77, SD = 2.98), F(1, 207) = 74.46, p<.001, η^2^ = .26, d = 1.47. This demonstrates that the priming worked sufficiently well for all target emotions, which were the most salient emotions in their respective condition.

### Outrage, Powerlessness, and Guilt

Given the fact that English speakers judge emotion words as having a similar meaning [58], the emotion words we had developed to measure outrage, guilt, and powerlessness were factor-analyzed to ensure the intended factor structure and dimensionality, and the factors’ distinctiveness. Based on the results of a principal component analysis, in accordance with Cattell’s [59] scree test it was decided to retain three factors in a subsequent exploratory factor analysis (EFA). As the expected dimensions of outrage, powerlessness, and guilt are not independent from each other, an oblique rotation method (oblimin) was chosen for the EFA. The three-factor solution of the EFA yielded acceptable results; all items fulfilled the simple structure criterion of only loading highly on one factor and low or significantly lower on the other factors. The expected factors emerged: outrage, powerlessness, and guilt. Which items belong to what factor is shown by the rotated factor pattern in Table 1. Confirmatory factor analyses and likelihood-ratio tests further showed a three-factor solution to be superior to a one- or two-factor solution, supporting the notion that these three factors are related but distinct.

**Table 1:** Rotated factor pattern (with oblique rotation) for the exploratory factor analysis over all emotion items, yielding three distinct factors labeled Outrage, Powerlessness, and Guilt.

	Outrage	Powerlessness	Guilt
Furious	0.77	−0.06	−0.02
Upset	0.56	0.04	0.07
Angry at others	0.54	−0.07	0.05
Offended	0.52	0.23	−0.34
Nervous	−0.07	0.74	−0.02
Helpless	0.10	0.70	0.01
Exposed	−0.05	0.57	0.05
Weak	0.03	0.51	0.21
Sorry	−0.10	0.00	0.73
Regret	0.09	0.07	0.66
Guilt	−0.01	0.16	0.65
Remorse	0.06	0.02	0.62

### Tests of Hypotheses

Reliable composite scores were created based on the three factors: outrage (Cronbach’s α = .72), powerlessness (Cronbach’s α = .75), and guilt (Cronbach’s α = .79). The only positive correlation between these three dependent variables was the one between powerlessness and guilt, r(212) = .46, p<.001. Outrage was neither significantly correlated with guilt, r(212) = −.11, p>.10, nor with powerlessness, r(212) = 0.10, p>.10. For each dependent variable two planned contrasts were carried out to test the predicted differences between humiliation, shame, and anger.

#### Outrage

In line with our prediction that levels of outrage should be similar for people who experienced humiliation and people who experienced anger, but higher than for people who experienced shame, the average intensity of reported outrage across the humiliation (M = 7.34, SD = 1.35) and anger (M = 7.57, SD = 1.14) conditions was significantly greater than the intensity of outrage reported in the shame condition (M = 7.19, SD = 1.51), F(1, 205) = 3.27, p<.05, η^2^ = .02, d = 0.28. A second pre-planned contrast found that the intensity of outrage did not differ reliably between the anger and humiliation conditions, F(1, 205) = 1.03, p>.05, η^2^ = .01, d = 0.17.

#### Powerlessness

Supporting our prediction that levels of perceived powerlessness should be similar for experiences of humiliation and shame, but higher than for experiences of anger, the average intensity of reported powerlessness across the humiliation (M = 5.77, SD = 1.79) and shame (M = 6.23, SD = 1.65) conditions was greater than the intensity of powerlessness reported in the anger condition (M = 4.68, SD = 2.23), F(1, 205) = 23.26, p<.01, η^2^ = .10, d = 0.69. A second pre-planned contrast found that the intensity of perceived powerlessness in the shame and humiliation conditions was not reliably different, F(1, 205) = 1.83, p>.05, η^2^ = .01, d = 0.24.

#### Guilt

In line with our prediction that levels of guilt should be lower for experiences of humiliation than for experiences of shame, the average intensity of reported guilt across the humiliation (M = 3.92, SD = 1.87) and anger (M = 3.15, SD = 1.89) conditions was significantly lower than the intensity of guilt reported in the shame condition (M = 5.76, SD = 1.7), F(1, 205) = 57.4, p<.001, η^2^ = .11, d = 1.17. The contrast between humiliated and angry people was also significant, F(1, 205) = 7.19, p<.01, η^2^ = .03, d = 0.44.

## Discussion

In this empirical investigation, we found that experiences of humiliation overlapped with those of anger and shame. Participants primed with humiliation reported relatively low levels of guilt and relatively high levels of other-directed outrage (like anger, but unlike shame); and relatively high levels of powerlessness (like shame, but unlike anger). Thus, we conclude that the emotional experience of “humiliation” is like that of “anger” in some respects, and like “shame” in others, but it is not the same as either one. Note that we do not claim to have discovered that humiliation is a discrete emotion, merely that the word appears to denote a commonsense category that evokes a particular configuration of emotional responses that partially overlap with, but are not identical to, anger and shame. As we discuss shortly, we believe that these findings may help advance the study of humiliation.

The reason why humiliation is similar to anger and shame in some respects might lie in the structure of humiliating situations. Perceived injustice characterizes both humiliation episodes [20], [23], [37]–[38], [44] and anger episodes (e.g., [60]–[62]), even when following moral transgressions [30]. Thus it is not surprising that for both anger and humiliation this perception of injustice then leads to low guilt and to high outrage (in contrast to shame). Humiliation episodes are also characterized, however, by powerlessness as a result of the publicly observable power asymmetry between ‘humiliator’ and ‘humiliatee’ inherent to humiliating situations (cf. [40]). It is precisely this quality, which differentiates humiliation from anger, that might prevent the outrage evoked by a humiliating situation from breaking out into aggression and violence. This might also explain the findings by Ginges and Atran [29].

There are a number of important implications of this research. First, although humiliation is often theoretically linked to violence due to the sense of injustice and outrage associated with it [27], [30], [43], Ginges and Atran [29] found that Palestinians who experienced injustice and felt humiliated as a Palestinian tended to be less likely, at least in the short term, to endorse political violence. Similarly, it was found that insults and offenses against someone’s honor – both characteristic of situations that are argued to share commonalities with humiliating situations – do not necessarily result in increased aggression or violence (e.g., [25]). Ginges and Atran [29] suggested that the humiliation-caused inertia, neither engaging in antisocial (e.g. violence) nor prosocial behavior (e.g. reconciliation), might be a consequence of the feeling of powerlessness that could accompany the outrage of humiliation (which otherwise, without the powerlessness, might have the same antisocial consequences anger often has). Our results support this suggestion and raise interesting possibilities regarding the particularly aversive characteristics of humiliation we suggested at the outset of this paper.

As a mix of outrage and powerlessness, the experience of humiliation may be associated with confused action tendencies. The feeling of other-directed outrage might lead to a desire to attack the source of injustice (the action tendency associated with anger; [63]–[65]). Yet, the feelings of powerlessness present in experiences of humiliation might lead to an action tendency of withdrawal (the action tendency associated with shame). Thus, people experiencing humiliation may have no simple way of acting to regulate this aversive state. This has significant clinical as well as social implications, and appears an important topic for future research, as is a broader investigation of how people manage to cope with states of humiliation. For example, Klein [1] suggests that humiliating events remain particularly vivid in the minds of victims, across time. The conflicting action tendencies associated with humiliation could explain this.

We note that we investigated the experience of humiliation in an intergroup context. This seems to us to be particularly important because humiliation is often associated with attacks against social identities. People frequently experience injustice because of their ethnicity, religion, or sexual orientation, as also evidenced by the memories participants reported in our study. Yet it is clear that people also experience intragroup humiliation, because, for example, they do not obey group norms (cf. [30]). It is a matter for future investigations to determine whether humiliation in intragroup contexts is experienced in a similar fashion. While our research moved beyond typical undergraduate populations, it was confined to a relatively small, well-educated Western sample, and comparisons of different social groups (e.g., Blacks vs. Hispanics, Muslims vs. Non-Muslims, heterosexuals vs. homosexuals) as well as cultures are sorely needed to determine the extent to which our results are variable across groups and cultures [66]–[67].

Our study focused on the affective dimensions of humiliation and how it compares on these dimensions to other emotions such as shame and anger. We believe future work is needed to investigate cognitive and motivational dimensions. For instance, appraisals that may give rise to a humiliated (as opposed to angry or ashamed) state. We might expect that, like angered people, humiliated people would regard another as being the cause of their experience, would appraise the cause of their experience as being unfair and would regard themselves as in the right. However, humiliated people are more likely to appraise themselves as being powerless in the situation than angered (or perhaps even ashamed) people. Thus, it may be that the relationship between humiliation and powerlessness is bidirectional: humiliation leads to feelings of powerlessness, but powerlessness might be associated with appraisals of an experience that lead to feelings of humiliation instead of anger. Future research may also use these findings as a starting point in investigations of behavioral consequences of humiliation. For example, while humiliation is often seen anecdotally as a cause of violence, empirical investigation suggests that humiliation leads to inertia rather than violence [29]. Whether a negative event such as an insult results in violence or inertia might depend on the chronic or situational levels of powerlessness of the insulted person. Someone high in power may appraise the situation in such a way that they experience anger and respond with violence, whereas someone low in power might appraise the same situation in such a way that they experience humiliation and respond with inertia.

In sum, we conclude that humiliation in an intergroup context is experienced as an emotional state with the following pattern of affective characteristics: intense other-directed outrage, low guilt, but intense feelings of powerlessness. This study is a first step into the investigation of humiliation and its impact on social life. The cognitive and motivational characteristics of humiliation, the long-term effects of humiliation at an individual and collective level, and the extent to which these findings are variable across social groups and cultures remain to be explored.
